# Can HIV incidence testing be used for evaluating HIV intervention programs? A reanalysis of the Orange Farm male circumcision trial (ANRS-1265)

**DOI:** 10.1186/1471-2334-10-137

**Published:** 2010-05-27

**Authors:** Agnès Fiamma, Pascale Lissouba, Oliver E Amy, Beverley Singh, Oliver Laeyendecker, Thomas C Quinn, Dirk Taljaard, Bertran Auvert

**Affiliations:** 1 Department of Medicine, University of California Los Angeles Program in Global Health, Johannesburg, South Africa; 2 Institut National de la Santé et de la Recherche Médicale, INSERM U1018, Villejuif, 94804, France; 3 Department of Medicine, Division of Infectious Diseases, The Johns Hopkins University School of Medicine, Baltimore, Maryland, 21205 USA; 4 National Institute of Allergy and Infectious Diseases, National Institutes of Health, Bethesda, Maryland, 20892, USA; 5 National Institute for Communicable Diseases, Sandringham, Johannesburg, 2131, South Africa; 6 Progressus Research and Development, Northcliff, Johannesburg, 2115, South Africa; 7 Hôpital Ambroise-Paré, Assistance Publique- Hôpitaux de Paris, Boulogne, 92100, France; 8 University of Versailles, 78280, Guyancourt, France

## Abstract

**Background:**

The objective of this study was to estimate the effect of male circumcision (MC) on HIV acquisition estimated using HIV incidence assays and to compare it to the effect measured by survival analysis.

**Methods:**

We used samples collected during the MC randomized controlled trial (ANRS-1265) conducted in Orange Farm (South Africa) among men aged 18 to 24. Among the 2946 samples collected at the last follow-up visit, 194 HIV-positive samples were tested using two incidence assays: Calypte HIV-EIA (BED) and an avidity assay based on the BioRad HIV1/2+O EIA (AI). The results of the assays were also combined (BED-AI). The samples included the 124 participants (4.2% of total) who were HIV-positive at randomization. The protective effect was calculated as one minus the intention-to-treat incidence rate ratio in an uncorrected manner and with correction for misclassifications, with simple theoretical formulae. Theoretical calculations showed that the uncorrected intention-to-treat effect was approximately  independent of the value of the incidence assay window period and was the ratio of the number tested recent seroconverters divided by the number tested HIV-negative between the randomization groups. We used cut-off values ranging from 0.325 to 2.27 for BED, 31.6 to 96 for AI and 0.325-31.6 to 1.89-96 for BED-AI. Effects were corrected for long-term specificity using a previously published formula. 95% Confidence intervals (CI) were estimated by bootstrap resampling.

**Results:**

With the highest cut-off values, the uncorrected protective effects evaluated by BED, AI and BED-AI were 50% (95%CI: 27% to 66%), 50% (21% to 69%) and 63% (36% to 81%). The corrections for misclassifications were lower than 50% of the number of tested recent. The corrected effects were 53% (30% to 70%), 55% (25% to 77%) and 67% (38% to 86%), slightly higher than the corresponding uncorrected values. These values were consistent with the previously reported protective effect of 60% (34% to 76%) obtained with survival analysis.

**Conclusions:**

HIV incidence assays may be employed to assess the effect of interventions using cross-sectional data.

## Background

Since the first detuned enzyme immunoassay (EIA) to detect recent HIV seroconversion was described in 1998 [1], there has been great interest in the application of laboratory methods to measure HIV incidence in cross-sectional samples. Currently, the most widely used incidence assay is the BED capture assay [2]. HIV incidence estimation is increasingly being incorporated into HIV/AIDS surveillance activities in both resource-rich and developing countries [3]. In 2005, the UNAIDS Reference Group on Estimates, Modeling and Projections issued a cautionary statement about using BED to estimate HIV incidence and called for the development of additional laboratory and modeling methodologies [4].

The ability to reliably measure HIV incidence using cross-sectional data has vast public health importance in HIV surveillance as well as in the preparation of cohorts for vaccine, microbicide, pre exposure prophylaxis or other potential HIV prevention studies. Reliable cross-sectional HIV incidence measures would reduce the need to recruit and maintain large and costly longitudinal cohorts. However, two of the current challenges in using HIV incidence assays to characterize HIV incidence are knowledge of the incidence assay window period (i.e. time interval during which individuals are characterized as recently infected) and misclassifications.

Another potential interest of HIV incidence testing is to conduct risk factor analysis on HIV incidence and to evaluate the effect of interventions aiming to reduce the spread of HIV. Here, the question is to estimate the ratio of incidence rates among subgroups of a given population. The objective of this study was to demonstrate the utility of HIV incidence assays in estimating the effect of an intervention. This was achieved by using two incidence assays applied to samples collected during the male circumcision (MC) randomized controlled trial (ANRS-1265) conducted in Orange Farm (Gauteng Province, South Africa) [5], and by comparing the results with those already published and obtained by classical survival analysis conducted on the same data.

## Methods

### Longitudinal data

The technical details of the Orange Farm MC trial (ANRS-1265) have been published elsewhere [5]. Briefly, male participants, aged 18 to 24, were recruited from the general population of the township of Orange Farm and followed up for 21 months. The recruitment, randomization between intervention and control groups, and follow-up were conducted independently of the HIV status of participants. At inclusion after providing written informed consent and during each follow-up visit V2, V3 and V4 at about 3, 12 and 21 months, a blood sample was obtained. The data used in the current study includes 596 additional follow-up visits, which were collected after the database used to report the results of the trial on HIV incidence [5] was locked, as described elsewhere [6]. The research protocol was reviewed and approved by the University of Witwatersrand Human Research Ethics Committee (Medical) on February 22nd, 2002 (protocol study no. M020104).

### Laboratory methods

All blood samples collected were tested for HIV using three enzyme immunoassays (EIA) as described previously [5]. Blood samples testing positive for HIV were retested using two HIV incidence assays: Calypte HIV-1 BED Incidence EIA (BED, Calypte Biomedical Corporation, Lake Oswego, Oregon, USA), and an avidity assay based on the BioRad HIV 1/2+O EIA (Avidity, Bio-Rad Laboratories, Redmond, Washington, USA). The BED assay was performed according to the manufacturer's protocol and gave a normalized optical density ranging between 0.034 to 3.9.

The avidity assay was performed as previously described [7]. Briefly, samples, placed in two consecutive wells, were diluted 1:10 and incubated at 4°C for 30 minutes for the initial antibody binding step. One of the wells for each sample was then incubated with 0.1M Diethylamine for 30 minutes at 37°C for the chaotropic disassociation step, while the other well was incubated with wash buffer. The avidity index (AI) was calculated as the optical density (OD) of the treated well divided by the OD of the untreated well multiplied by 100. AI varied from 5.7 to 131.6. Values greater than 100 were reported as 100. There were six individual time points with untreated avidity OD values lower than the avidity OD threshold (0.1). These individuals were considered as recently infected.

### Data analysis

The details of the methods used are given in the Additional file 1.

Samples yielding a result lower or equal to the cut-off value for an incidence assay were reported as "tested recent seroconverters". All other HIV-positive participants were reported as "tested long-term seroconverters" for the assay. The two assays were also used in combination (BED-AI). In this case, participants with both BED and AI results lower or equal to the corresponding cut-off values were reported as "tested recent seroconverters". Uncorrected results were calculated without correction for misclassifications. Corrected results were calculated by correcting for misclassifications. We used the conventional cut-off values of 0.80 for BED and 40 for AI. We also varied the cut-off values in order to obtain window periods of 3, 6, 9, 12, 15 and 18 months for both assays from samples of the 67 participants who became HIV positive during follow-up.

Using the results given by the two assays, we calculated the window periods with conventional cut-offs for each assay, as well as the cut-off values for the two assays in order to obtain predetermined window periods of 3, 6, 9, 12, 15 and 18 months. We statistically compared the results (recent/not recent) given by the BED and the AI assays when using the conventional cut-off values. We estimated the window periods when combining the two assays. We calculated the proportion of false long-term seroconverters and the proportion of false recent seroconverters. For each individual, we assessed the variations of the assay results over time and, finally, the effect of MC on HIV incidence, which is the main outcome of this study. Confidence intervals were obtained using the bootstrap method with 1000 repetitions, except for the confidence intervals of proportion calculated using Bayesian estimation [8]. As shown in Additional file 1, the uncorrected and corrected incidence rate ratios (IRR) at a given point in time were calculated using the following formulae:(1)

and(2)

in which N_TR _is the number of tested recent seroconverters, N_- _is the number of HIV negative participants, N_+ _is the number of HIV positive participants and is the long-term specificity. The uncorrected IRR is independent of the window period. The corrected IRR depends on the long-term specificity, which varies with the window period (see below).

Among the 3153 trial participants who had a HIV test at inclusion and at least one HIV test during follow-up, 2946 participants were tested for HIV, BED and AI at V4. Data from these participants were used to calculate the effect of the intervention. Among them, 194 tested HIV-positive at V4, 124 having tested HIV-positive at randomization and 70 becoming HIV-positive during follow-up.

Among the 3153 participants, 72 participants became HIV-positive during follow-up but five had a missing follow-up visit immediately preceding the follow-up visit when they tested HIV-positive for the first time. The remaining 67 participants were used to calculate the window periods corresponding to specified cut-off values and to calculate cut-off values corresponding to specified window periods.

## Results

### Window periods with conventional cut-offs

Using the 67 participants who became HIV positive during follow-up, the number of tested recent seroconverters was found to be 47 for BED and 37 for AI when using the conventional cut-off values. The corresponding window periods were 185 days (6.0 months; 95% CI: 146 to 227) and 135 days (4.4 months; 95% CI: 101 to 176), respectively.

### Cut-offs for various predetermined window periods

Using the same 67 participants who became HIV positive during follow-up, the cut-off values corresponding to predetermined window periods of 3, 6, 9, 12, 15 and 18 months for each of the two assays were calculated and are given in Table 1.

**Table 1:** Cut-off values for BED and AI incidence assays for predetermined incidence assay window periods

Window period	3 m (91.5 d)	6 m (183 d)	9 m (274 d)	12 m (366 d)	15 m (457 d)	18 m (549 d)
BED Cut-off	0.325	0.733	0.90	1.51	1.89	2.27
95% CI	0.21 to 0.41	0.49 to 1.14	0.66 to 1.50	0.88 to 1.89	1.58 to 2.17	2.02 to 2.64
AI Cut-off	31.6	80.5	90.0	94.0	96.0	NC
95% CI	20.6 to 37.0	42.9 to 90.1	84.0 to 94.0	90.0 to 97.5	94.0 to 99.7	

m: months

d: days

AI: avidity index

NC: Not calculable

CI: Confidence interval

### Window periods when combining the two assays

The incidence assay window periods obtained when combining the two assays are indicated in Table 2 for each of the five pairs of cut-of values. As expected, because of the discordance between the results given by the two tests, the window period for each pair was slightly shorter than the corresponding window period for each test taken individually.

**Table 2:** Incidence assay window periods obtained when combining BED and AI incidence assays for various pairs of cut-off values

BED - AI Cut-off	0.325 - 31.6	0.733 - 80.5	0.90 - 90.0	1.51 - 94.0	1.89 - 96.0
Window period	65.9 d	159 d	213 d	268 d	300 d
95% CI in days	45.3 to 93.5	122 to 211	163 to 264	211 to 352	234 to 388

d: days

AI: avidity index

CI: Confidence interval

### False long-term seroconverters

The proportion of false long-term seroconverters for each test used separately and in combination are indicated in Tables 3 and 4. It appears that this proportion, and consequently the sensitivity, was fairly independent of the cut-off values; sensitivity stayed above 75% when the assays were used independently or in combination.

**Table 3:** False recent seroconverters and protective effect of MC on HIV incidence as calculated using BED and AI separately

BED						
Cut-off	0.325	0.733	0.900	1.51	1.89	2.27
Window period	3m (91.5d)	6m (183d)	9m (274d)	12m (366d)	15m (457d)	18m (549d)
False LTS (%)	0/3 (0)	1/12 (8.3)	5/21 (23.8)	5/32 (16)	2/34 (5.9)	3/47 (6.4)
95% CI	0 to 0.53	0.5 to 32	9.6 to 44	6 to 30	1 to 17	2 to 16
Observed false RS (%)	1/124 (0.8)	6/124 (4.8)	8/124 (6.5)	NC	NC	NC
95% CI	0.05 to 3.8	1.9 to 9.6	3 to 12			
^1^Calculated false RS (%)	2.1	4.3	6.4	8.5	10.7	12.8
Tested RSs						
Intervention - Control	6 - 10	12 - 27	15-30	23 - 48	33 - 62	42 - 79
Uncorrected effect (%)	43	58	53	55	49	50
95% CI	-57 to 84	19 to 80	13 to 77	26 to 75	23 to 67	27 to 66
^2^Correction						
Intervention (%)	28	29	34	30	26	24
Control (%)	24	18	24	20	20	18
Total (%)	25	21	28	23	22	21
Corrected effect (%)	46	63	59	60	53	53
95% CI	NC	22 to 88	9 to 86	25 to 80	25 to 73	30 to 70
**AI**						
Cut-off	31.6	80.5	90.0	94.0	96.0	NC
Window period	3m (91.5d)	6m (183d)	9m (274d)	12m (366d)	15m (457d)	18m (549d)
False LTS (%)	0/3 (0)	1/12 (8.3)	3/21 (14)	4/32 (13)	4/34 (12)	NC
95% CI	0 to 53	0.5 to 32	4 to 33	4 to 27	3.8 to 25	
Observed false RS (%)	0/124 (0)	3/124 (2.4)	15/124 (12)	NC	NC	NC
95% CI	0 to 2.4	0.6 to 6.2	7 to 19			
^1^Calculated false RS (%)	2.9	5.9	8.8	11.8	14.7	NC
Tested RSs						NC
Intervention - Control	3 - 11	10 - 19	18-31	26 - 44	33 - 62	
Uncorrected effect (%)	74	50	45	44	50	NC
95% CI	19 to 100	-6 to 78	3 to 72	17 to 71	21 to 69	
^2^Correction						NC
Intervention (%)	77	47	39	36	36	
Control (%)	30	35	32	31	27	
Total (%)	40	39	35	33	30	
Corrected effect (%)	92	59	50	49	55	NC
95% CI	14 to 100	-14 to 100	-13 to 83	4 to 78	25 to 77	

LTS: Long-term seroconverters

RS: Recent seroconverters

m: months

d: days

AI: Avidity index

NC: Not calculable

CI: Confidence interval

^1^Estimated by linear regression (see Additional file 1)

^2^Correction for intervention and control = [N_HIV+_(1-False RS)]/N_tested recent_

**Table 4:** False recent seroconverters and protective effect of MC on HIV incidence as calculated using BED and AI in combination

BED-AI						
BED - AI Cut-off	0.325 - 31.6	0.733 - 80.5	0.900 - 90.0	1.51 - 94.0	1.89 - 96.0	NC
Window period	65.9d	159d	213d	268d	300d	NC
False LTS (%)	NC	1/9 (11)	1/13 (7.7)	2/16 (13)	3/27 (11)	NC
95% CI		1 to 40	0.4 to 30	2 to 33	3 to 26	
Observed false RS (%)	0/124 (0)	2/124 (1.6)	2/124 (1.6)	6/124 (4.8)	NC	NC
95% CI	0 to 2.4	0.25 to 5.0	0.3 to 5	1.9 to 9.6		
^1^Calculated false RS (%)	0.9	2.1	2.7	3.5	3.9	NC
Tested RSs						NC
Intervention - Control	2 - 7	8 - 17	9-21	11 - 30	16 - 41	
Uncorrected effect (%)	73	55	59	65	63	NC
95% CI	NC	-1 to 84	8 to 84	33 to 85	36 to 81	
^2^Correction						NC
Intervention (%)	36	21	24	25	20	
Control (%)	15	14	15	13	11	
Total (%)	19	16	17	17	13	
Corrected effect (%)	80	59	64	70	67	NC
95% CI	NC	-2 to 93	6 to 92	39 to 90	38 to 86	

See table caption of Table 3

### False recent seroconverters

The observed proportion of false recent seroconverters for each test used separately and in combination is indicated in Tables 3 and 4. This proportion increased with cut-off values, implying that long-term specificity decreases with increased cut-off values. The proportion of false recent seroconverters was similar between assays when compared for the same window period. Comparing the proportion obtained when combining the two assays with the assays used individually is meaningful because of the difference between window periods. Nevertheless, Tables 3 and 4 show that this proportion was slightly lower when combining the two assays. For example combining BED and AI with a window period of 268 days gave a proportion of false recent seroconverters of 4.8%, whereas using both BED and AI separately for a similar window period (274 days) gave a result of 6.4% and 8.8%, respectively. When using the conventional cut-off value for BED, the long-term specificity was 93.6% (1 minus 6.4%).

### Individual response of test results over time

The average (standard deviation) slope of the variation of assay results with time was 0.023 (0.027) per month for BED and 0.47 (1.08) per month for AI. The proportion of individuals with values given by the assays decreasing with time (i.e.: negative slope) was 15/104 (0.14; 95% CI: 0.086 to 0.22) for BED and 35/104 (0.34; 95% CI: 0.25 to 0.43) for AI. The proportion of individuals having values given by the two tests decreasing was 9/104 (0.087; 95% CI: 0.043 to 0.15). The proportion of those having values given by at least one test decreasing was 43/104 (0.41; 95% CI: 0.32 to 0.51).

### Comparison of results given by the BED and AI assays, and with HIV testing

We compared those tested recent seroconverters by the BED assay with those identified by AI when using the cut-off value of 0.73 for the BED assay and 80.5 for AI, which both correspond to a window period of six months. Among all 194 participants testing HIV-positive at V4, the BED assay and AI detected 39 and 29 seroconverters, respectively. Of these, 25 were tested recent for both assays, 151 were tested not recent for both assays, 14 were tested recent with BED and tested not recent with AI, and four were tested recent with AI and tested not recent with BED. The difference was statistically significant (p = 0.031; McNemar's test) and the kappa was 0.68 (95% CI: 0.54 to 0.82) indicating a *substantial *agreement between the two assays.

The comparison between the results given by the BED and AI assays with observed HIV incidence cases obtained from HIV testing is given in Table 5. This table shows that the agreement was *moderate *for a window period of nine months and became *fair *for window periods of 12 and 18 months. This table shows that the overall specificity decreases when the window period increases.

**Table 5:** Comparison of the results given by the BED and the AI assays with observed HIV incidence cases obtained from HIV testing

Visit A				
Label	V1	V3	V1	V2
HIV positive cases	127	148	124	124
HIV negative cases	11	35	32	56
Visit B				
Label	V2	V4	V3	V4
HIV positive cases	138	183	156	180
^1^HIV negative cases	0	0	0	0
From visit A to visit B				
Average duration	3.6 m	8.8 m	12.6 m	18.0 m
HIV incident cases	11	35	32	56
BED				
Cut-off	0.325	0.90	1.51	2.27
Window period	3.0 m	9.0 m	12.0 m	18.0 m
Tested recent	12	43	54	110
Tested recent not incident case	6	25	21	53
Tested recent and not incident case	6	18	33	57
Not tested recent and incident cases	5	10	11	3
Kappa statistics [95% CI]	0.64 [0.41 to 0.81]	0.55 [0.40 to 0.69]	0.31 [0.16 to 0.46]	0.39 [0.26 to 0.51]
Sensitivity (%) [95% CI]	55 [28 to 79]	71 [56 to 84]	66 [49 to 80]	95 [87 to 99]
Specificity (%) [95% CI]	95 [91 to 98]	88 [82 to 92]	73 [65 to 81]	54 [45 to 63]
AV				
Cut-off	31.6	90	94	NC
Window period	3.0 m	9.0 m	12.0 m	
Tested recent	11	49	44	
Tested recent and incident case	8	28	20	
Tested recent and not incident case	3	21	24	
Not tested recent and incident cases	3	7	12	
Kappa statistics [95% CI]	0.48 [0.31 to 0.64]	0.57 [0.43 to 0.71]	0.38 [0.23 to 0.53]	
Sensitivity (%) [95% CI]	73 [45 to 92]	80 [65 to 91]	63 [46 to 78]	
Specificity (%) [95% CI]	98 [94 to 99]	86 [79 to 91]	81 [73 to 87]	
BED-AI				
Cut-off	^2^0.40 - 41	1.51 - 94	NC	NC
Window period	9.0 m	8.8 m		
Tested recent	10	38		
Tested recent and incident case	7	27		
Tested recent and not incident case	3	11		
Not tested recent and incident cases	4	8		
Kappa statistics [95% CI]	0.70 [0.54 to 0.87]	0.68 [0.53 to 0.82]		
Sensitivity (%) [95% CI]	64 [36 to 86]	77 [62 to 89]		
Specificity (%) [95% CI]	98 [94 to 99]	93 [88 to 96]		

m: month

CI: Confidence interval

NC: Not calculable

AI: Avidity index

^1^For this analysis, the data was reduced to those being HIV positive at V4

^2^Obtained by linear interpolation from the data presented in Table 2

### Effect of MC on HIV incidence

We used the 2946 participants with HIV data at V4. Among these participants, 124 (4.2%) were HIV-positive at randomization, 194 were HIV-positive at V4 (114 in the intervention group and 80 in the control group) and 2752 were HIV-negative at V4 (1412 in the intervention group and 1340 in the control group). Using the samples collected at this fourth visit, the uncorrected and corrected protective effects of MC derived when using BED, AI and BED-AI for the six window periods are presented in Tables 3 and 4, when they were calculable. These tables indicate the number of tested recent seroconverters observed for each assay per intervention group, which, as expected, increased with the cut-off values. These tables also indicate the relative correction applied to the number of tested recent seroconverters to estimate the real number of recent seroconverters.

When the cut-off values were low, the small number of tested recent seroconverters led to lower statistical power, larger confidence intervals and imprecise estimation of the effect of MC on HIV incidence. When the cut-off values were increased, the uncorrected IRR tended to become closer to the HIV prevalence ratio which was 0.684 (80/114), corresponding to an effect of the intervention of 31.6%.

Tables 3 and 4 show that the corrections for misclassifications were in total always lower than 50% of the number of tested recent seroconverters. The highest cut-off values used for BED, AI and BED-AI were 2.27, 96 and 1.89-96, respectively. For each of these values, we obtained a total number of tested recent seroconverters of 121, 95 and 57, which in two cases was higher than the number (70) of participants who became HIV positive during the follow-up period. These cut-off values corresponded to uncorrected effects of the intervention for BED, AI and BED-AI of 50%, 50% and 63%, and corresponding corrected effects of 53%, 55% and 67%. These values obtained for the highest values of the cut-off are in reasonable agreement with the value of 60% (95% CI: 34% to 76%) obtained by survival analysis applied to the full dataset [5].

When recalculating the effect using only the 2684 participants who were HIV-negative at V3 and were followed up between V3 and V4, we obtained, using survival analysis, a HIV incidence rate of 0.010/person-year (py) (10/989.8/py among the intervention group and 0.026/py (25/965.2/py) among the control group. These incidence rates led to a protective effect of 61.0% (95% CI: 21.0% to 82.7%). The uncorrected and corrected effects obtained for BED, AI and BED-AI with cut-off values of 0.900, 90.0 and 1.51-94.0, which corresponded to window periods similar to the period between V3 and V4 are indicated in Tables 3 and 4. These values are in reasonable agreement with the value obtained by survival analysis (see above).

When considering the results obtained for the various window periods, it can be noted that a) the uncorrected effects of the intervention are always lower than the corresponding corrected effects, and b) the results tend to become more significant as the cut-off value is increased. Tables 3 and 4 do not provide evident argument for preferring one assay over the other, and show that using the two assays in combination is not obviously more advantageous than using the assays independently. However, it can be noted that the BED assay allows the use of a cut-off value corresponding to a window period longer than the longest window period given by the AI assay.

## Discussion

Using a longitudinal dataset obtained during the first MC randomized controlled trial [5], we were able to demonstrate for the first time that the effect of the intervention could have been approximately estimated by HIV incidence testing applied to blood samples collected at the final follow-up visit. This result was obtained despite the presence of a substantial proportion of individuals who were HIV-positive at recruitment. Such results imply that HIV interventions may be assessed using HIV incidence assays on samples obtained from a cross-sectional survey by calculating incidence rate ratios. In addition, the method we have used is relatively simple because it does not require the precise knowledge of the window period.

This study has several limitations. It is known that those on antiretroviral treatment should be excluded from BED testing to improve the predictive value of detecting recent infections [9,10]. This was not done in the present study because such information was not available. However, it is likely that only a very small number of participants were receiving anti-retroviral (ARV) therapy because, in contrast with the current situation in this community, ARVs were not widely available during the course of the study. Moreover, because only a few participants were aware of their HIV status and because of their young age, the proportion of HIV-positive participants eligible for ARVs is expected to have been low. Another limitation is that the correction required the knowledge of long-term specificity. This value was calculated using the same data set and thus was not independent. Finally, this study provides some information about the comparative behavior of the assays when used on the same samples but did not have the power to formally compare the results given by these assays a) between them and b) with the results obtained when using survival analysis applied to the exact seroconversion data. This lack of power is evidenced by the wide confidence intervals of the estimated effects. Further studies are needed to compare these assays and to evaluate the benefit of using them in combination.

To our knowledge, the Orange Farm MC study is the only randomized controlled trial to have recruited and followed participants irrespective of their HIV status. Hence, the results obtained in this study may be difficult to reproduce. However, this allowed us to simulate what would happen in cross-sectional studies where HIV positive participants are not excluded.

Our results were obtained from young men (18 to 24 years old) in an area of relatively high HIV prevalence. According to the 2008 South African National HIV Prevalence, Incidence, Behavior and Communication Survey, HIV prevalence among young men 20 to 24 was 5.1% and 21.1% among women of the same age [11]. Among young men in the Orange Farm area, as in South Africa, HIV prevalence is relatively low and HIV incidence is high. These characteristics may have facilitated this study. With higher HIV prevalence, the impact of corrections for misclassifications would have been higher and may have reduced the precision of the estimations [12,13]. However, we think that the use of HIV incidence testing to assess the effect of interventions through HIV incidence rate ratios can be used as long as the sample size is adapted to the long-term specificity of the assays and when the cut-off values are such that the corrections for misclassifications are kept reasonably low.

In this study, we obtained similar results for each assay, and combining the assays did not noticeably improve the results. A study conducted using blood samples collected among female sex workers in the Dominican Republic found a good correlation between BED and AI methods (100%; kappa = 1.0) using an unweighted kappa statistic in pairwise comparisons [14]. Our study showed a lower agreement between the two assays.

The choice of the cut-off values is important to obtain a precise estimate of the incidence rate ratio. The cut-off values must be high enough to yield a high number of tested recent seroconverters and thus a small confidence interval, but low enough to yield a low number of false recent seroconverters and thus a small correction for misclassifications. The cut-off values should also be high enough to correspond to a reasonably large window period, in order to smooth the measured effect on an adequately large time interval. Our study shows that cut-off values corresponding to window periods of 12-18 months can be used in this population of young men.

The long-term false-positive ratio given by the BED assay with a cut-off value of 0.80 was estimated to be 0.0169 among a sample of 4869 South African individuals [15]. Our estimate was slightly higher with a similar cut-off value. It was closer to the value of 0.052 estimated from plasma samples collected in Zimbabwe [16].

Some studies have highlighted the difficulties of using incidence testing for estimating HIV incidence [17-19]. The first issue is the need to know the window period corresponding to a given assay cut-off. The second is that the misclassification may vary with factors such as age, gender, HIV subtype, ARV coverage, stage of the epidemic, population and region [17]. The interest in correction procedures that have been proposed to correct HIV incidence rates derived from cross-sectional surveys of biomarkers has been discussed [13,20]. In contrast, some studies found a good concordance in the classification of recent HIV-1 infections between incidence testing and in the correlates of acquisition of infection [21]. HIV incidence testing has been used in some studies to assess trends and risk factors for HIV incidence [22,23]. The value of the window period for the BED assay obtained in this study using a cut-off value of 0.8 was close to the value of 187 days estimated using longitudinal data collected among 85 South-African women [16].

In the current study, we did not need to estimate HIV incidence nor did we need to know the window period precisely. Indeed, the effect of the intervention was assessed through an incidence rate ratio, which can be estimated using incidence testing without knowing the value of the window period, as shown by the uncorrected formula that we have used. This formula depends mainly on the estimation of the ratio of the number of recent seroconverters between the intervention and control groups. The use of the corrected formula required the knowledge of the long-term specificity, which varies with the window period. Because this term was used to calculate a correction, which can be substantial, a good estimation of its numerical value is required.

The correction that we have used is based on a formula published by McDougall and colleagues [24]. This formula assumes that, among those who survive, the number of HIV infections having occurred is the same during the window period than in the period preceding the window period and of the same duration. In a situation where HIV prevalence is rising and when using a window period longer than conventionally used, this assumption may no longer hold.

Vaccine, microbicide, pre exposure prophylaxis or other potential HIV prevention or intervention studies would benefit from using more precise and recent comparison of incidences through the estimation of incidence rate ratios derived from cross-sectional surveys. Cross-sectional HIV incidence rate ratio estimations would reduce the need to recruit large longitudinal cohorts, which are costly, and may suffer from recruitment bias. Using cut-off values higher than conventionally employed and appropriate age-groups, when possible, would obviate the need to recruit large numbers of people such as what was previously done in Uganda for example, where a cross-sectional survey of more than 21 000 people was evaluated with a BED cut-off value of 0.4 [25].

Estimating the effect of an intervention by assessing the incidence rate ratio is important in HIV prevention research. Conducting randomized controlled trials and following up HIV-negative participants to measure HIV incidence among various groups and then comparing HIV incidence by calculating incidence rate ratio is not always feasible, sometimes for ethical reasons. For example, in conducting intervention studies, the effect of the intervention may not be assessed using longitudinal cohorts but could only be measurable using cross-sectional studies. The current MC roll-out study in Orange Farm in which MC is being made available to the community (ANRS-12126) with the objective of testing the effect of MC on HIV incidence in 'real life' is an example. The results of this study show that this effect may well be assessed by conducting a post-intervention cross-sectional study. One additional advantage is that cross-sectional respondents are more likely to be representative of the population. Such characteristics are difficult to obtain when recruiting a cohort. Another example is Project Accept (National Institute of Mental Health, HPTN 043), a community randomized trial providing community mobilization, mobile HIV voluntary counseling and testing and comprehensive post-test supportive services. In this trial, the outcome will be assessed using HIV incidence assays to estimate HIV incidence rate ratio in order to compare control and intervention communities.

## Conclusions

HIV incidence assays may be employed to assess the effect of interventions using cross-sectional data.

## Competing interests

The authors declare that they have no competing interests.

## Authors' contributions

AF and BA conceived of the study and wrote the first draft. All authors critically commented on the paper, read and approved the final manuscript.

## Financial support

Supported by ANRS-1265 and ANRS-12126, Agence Nationale de Recherches sur le Sida et les hépatites virales (France), the US National Institute of Mental Health as a cooperative agreement, through contracts U01MH066687 (Johns Hopkins University), U01MH066688 (Medical University of South Carolina), U01MH066701 [University of California, Los Angeles (UCLA)], and U01MH066702 (University of California, San Francisco). In addition, this work was supported by the HIV Prevention Trials Network (HPTN Protocol 043) of the Division of AIDS of the US National Institute of Allergy and Infectious Diseases and by the Office of AIDS Research of the US National Institutes of Health. Additional support provided by the HIV Prevention Trials Network (HPTN) sponsored by the NIAID, National Institute on Drug Abuse (NIDA), National Institute of Mental Health (NIMH), and Office of AIDS Research, of the NIH, DHHS (U01-AI-068613) and with the support of IFAS, the French Institute of South Africa (UNMIFRE CNRS n°25). Additional support was provided by the Division of Intramural Research, NIAID.

## Pre-publication history

The pre-publication history for this paper can be accessed here:

http://www.biomedcentral.com/1471-2334/10/137/prepub

## Supplementary Material

Additional file 1 **Annex 1**. This file contains the details of the methods. Click here for file
