# Supplementary material for: Can HIV incidence testing be used for evaluating HIV intervention programs? A reanalysis of the Orange Farm male circumcision trial (ANRS-1265)
Source: BMC Infect Dis. 2010 May 27;10:137. doi: 10.1186/1471-2334-10-137 (PMC2890001; doi:10.1186/1471-2334-10-137)
Supplement: Additional file 1 — Annex 1. This file contains the details of the methods. [file 1471-2334-10-137-S1.PDF]

ANNEX 1

Incidence assay window periods with conventional cut-offs

To estimate the incidence assay window period (W) for each assay, we included all participants who became HIV positive during follow-up. Using the value given by each assay when participants were first tested HIV-positive, we calculated for each individual (i) the probability P_i that he was in the window period by using the time interval (D_i) between the last HIV-negative test and the first HIV-positive test. P_i was calculated by the following formula limited by 1:

$$P_i = \frac{W}{D_i} \qquad \text{(Formula 1)}$$

For example, with W=180 days, an individual being HIV-negative at V1 and HIV-positive at V2, 252 days later, would have a probability of 0.71 (=180/252).

A window period was then estimated for a given cut-off by the value of W such that the sum of the values P_i equals the number (N_{TR}) of tested recent seroconverters.

The probability of observing the result given by the assay can be zero. This was the case for participants tested not recent by BED or AI who had seroconverted during the window period. These zero values prevented the use of the method of maximum likelihood to estimate W. Nevertheless, when omitting participants with zero values, the two methods gave consistent results, with difference between estimations of the window period for each assay less than 2%.

Cut-offs for various predetermined window periods

To estimate the cut-off values for the two assays in order to obtain predetermined window periods of 3, 6, 9, 12, 15 and 18 months, we used the data from the 67 participants who became HIV positive during follow-up. For predetermined window periods of 3 and 6 months, we calculated Pi for each individual as indicated above (Formula 1). The cut-off value was then determined in order to obtain a number of tested recent seroconverters equal to the sum of the values Pi. For predetermined window periods of 9, 12, 15 and 18 months, we used the value given by each assay when participants were tested HIV-positive at the last visit, and Pi was then calculated as follows: using the interval Li between the last time participants were tested HIV negative and the first time participants were tested HIV positive, we used the following formula limited by 0 and 1:

$$P_i = \frac{W - L_i}{D_i} \qquad \text{(Formula 2)}$$

For example, with W=365 days, an individual being HIV-negative at V1 and HIV-positive at V2, 252 days later, and at V3, 275 days after V2, would have a probability of 0.36 (=365-275)/252).

Window periods when combining the two assays

To estimate the window periods when combining the two assays, we used all those who seroconverted during follow-up. To identify recent seroconverters, we used the five pairs of cut-off values corresponding to the identical window periods (3, 6, 9, 12, 15 and 18 months) when each assay was taken separately. The window periods for each pair of cut-off values were determined with the method indicated above to estimate the window period of each test, except that Pi was calculated using Formula 1 for the first two pairs and using Formula 2 for the last three pairs of cut-off values.

False long-term seroconverters
The true long-term seroconverters are those with an HIV infection having occurred outside the window period and tested not recent. We estimated the proportion of false long-term seroconverters as the proportion of those with a value given by BED, AI or BED-AI higher than the cut-off (i.e. tested not recent) among those who became HIV-positive during a time interval between the last HIV-negative test and the first HIV-positive test shorter than the window period. The sensitivity was calculated as one minus this proportion.

False recent seroconverters among those with long-term infection
The true recent seroconverters are defined as those with an HIV infection having occurred within the window period and tested recent. We estimated the proportion of false recent seroconverters among those with long-term infection as the proportion of those with a value given by BED, AI or BED-AI lower or equal to the cut-off (i.e. tested recent) among those who became HIV-positive during a time interval between the last HIV-negative test and the first HIV-positive test greater than twice the window period [1]. The long-term specificity (ρ_2), which was used to correct the window period (see above), was calculated as one minus this proportion. For a window period shorter than 10.5 months, we estimated this proportion among the 124 participants who were HIV-positive at recruitment with a blood sample obtained at the last follow-up visit (V4), about 21 months later. For a window period longer than 10.5 months, we calculated the long-term specificity by linear extrapolation.

Individual response of test results over time
To assess the variations of the assay results over time, we calculated for each individual who was HIV-positive at recruitment the mean-square slope of the regression line fitted to the test results as a function of time. This slope is supposed to be positive because the results obtained with the tests are meant to increase over time. For each assay used separately, we calculated the average slope of these regression lines and the proportion of individuals with a negative slope. For this analysis, we used the 104 HIV-positive individuals with a blood sample obtained at recruitment and at each of the three follow-up visits.

Comparison of results given by the BED and AI assays, and with HIV testing
To compare the results (recent / not recent) given by the BED and the AI assays on an individual blood sample, we used the McNemar's test. Concordance between the two assays was assessed using the kappa statistic [2].

To compare the results given by the BED and AI assays with observed HIV incident cases obtained from HIV testing, we selected the individuals who were HIV-positive at V4 and who had an HIV-test at V3. The number of individuals who seroconverted for HIV during this period was compared with the number of those who tested recent with the BED and AI assays used separately with a cut-off value corresponding to a window period of 9 months using the kappa statistic. This analysis was repeated using the data between V1 and V2, V3 and V4, V1 and V3, and V2 and V4, separated by about 3 months, 9 months, 12 months and 18 months, respectively.

Effect of MC on HIV incidence

To assess the effect of MC on HIV acquisition, we used the blood samples collected at the last follow-up visit (V4). By doing so, we simulated a situation in which the effect of an intervention is measured using a cross-sectional survey conducted at the end of an intervention program. Consequently, we used all the samples collected among those for which a blood sample was available at V4. These samples corresponded to participants who were either HIV-positive at recruitment (124, 4.2%), or who became HIV positive (70, 2.4%), or who remained HIV-negative during follow-up (2752, 93.4%).

The HIV incidence rate (IR) was obtained using the following consensus formula [3]:

$$IR = \frac{N_{TR}\frac{365}{W}}{N_{-} + \frac{N_{TR}}{2}\frac{365}{W}}$$ in which N_{-} is the number of HIV negative participants.

It follows that the uncorrected HIV incidence rate ratio (IRR) was calculated by

$$IRR_{uncorrected} = \frac{\left(\frac{N_{TR}\frac{365}{W}}{N_{-} + \frac{N_{TR}}{2}\frac{365}{W}}\right)_{\text{Intervention group}}}{\left(\frac{N_{TR}\frac{365}{W}}{N_{-} + \frac{N_{TR}}{2}\frac{365}{W}}\right)_{\text{Control group}}} \quad \text{(Formula 3)}$$

which can be approximated by $IRR_{uncorrected} = \frac{\left(\frac{N_{TR}}{N_{-}}\right)_{\text{Intervention group}}}{\left(\frac{N_{TR}}{N_{-}}\right)_{\text{Control group}}}$ (Formula 4).

Indeed, with the numerical values reported in this study, especially the much higher number of participants testing HIV-negative in comparison with the number of those tested as recent seroconverters, the relative difference between the IRR given by formulae 3 and 4 did not exceed 1.5%. We calculated the effect of the intervention by 1-IRR. The main advantage of Formula 4 is that the calculation of the IRR does not depend on the window period. These formulae show that when the cut-off values are increased, the number of recent seroconverters tends to the total number of HIV-positive and the IRR tends to become closer to the HIV prevalence ratio.

The corrected number of recent seroconverters can be calculated from the number of those tested recent seroconverters by the formula given by McDougall and colleagues [1]. It follows that:

$$IRR_{corrected} = \frac{\left(\frac{N_{TR} - N_{+}(1-\rho_2)}{\Omega N_{-}}\right)_{\text{Intervention group}}}{\left(\frac{N_{TR} - N_{+}(1-\rho_2)}{\Omega N_{-}}\right)_{\text{Control group}}}$$

In the above equation, Ω, is a function of the sensitivity, long-term and short-term specificity [1], or, as shown by Welte and colleagues [4], can be considered only as a function of the long-term specificity. In any case, it can be assumed that Ω is the same in each randomization group. It follows that:

$$IRR_{corrected} = \frac{\left(\dfrac{N_{TR} - N_{+}(1-\rho_2)}{N_{-}}\right)_{\text{Intervention group}}}{\left(\dfrac{N_{TR} - N_{+}(1-\rho_2)}{N_{-}}\right)_{\text{Control group}}}$$

The above correction consists of subtracting $N_{+}(1-\rho_2)$ from N_{TR} in each group to take into account the long-term specificity, which is one minus the proportion of false recent seroconverters among those with long-term infection.

The relative correction applied to N_{TR} in each group is $\frac{N_{+}(1-\rho_2)}{N_{TR}}$. The number of tested recent seroconverters and the relative correction increased with cut-off values. Because of these increases, for each assay used separately and in combination, we selected the higher cut-off value giving a relative correction of the number of tested recent seroconverters lower than 50% in the intervention group and in the control group. This value of 50% was an a priori choice. For these cutoffs values (one cut-off for BED, one cut-off for AI, and one pair of cut-offs for BED-AI) we calculated the uncorrected and corrected effects of the intervention, which were the main results of this study. These effects were qualitatively compared with the value obtained by survival analysis using the full data set, which was 60% (95%CI: 34% to 76%) [5]. Because we used window periods varying from 3 to 18 months, which are shorter than the total follow-up of about 21 months, the effects obtained using the incidence assays were also compared with the effect obtained by classical survival analysis when using only the data collected between V3 and V4, which were separated by about 9 months.

REFERENCES

1. McDougal JS, Parekh BS, Peterson ML, Branson BM, Dobbs T, Ackers M, Gurwith M: **Comparison of HIV type 1 incidence observed during longitudinal follow-up with incidence estimated by cross-sectional analysis using the BED capture enzyme immunoassay**. *AIDS Res Hum Retroviruses* 2006, **22**(10):945-952.
2. Landis JR, Koch GG: **A one-way components of variance model for categorical data**. *Biometrics* 1977, **33**:671-679.
3. **BED-CEIA Incidence and Adjustment Formula**. 2006(http://www.cdc.gov/nchstp/od/gap/docs/surveillance/BED-CEIA%20Incidence%20and%20Adjustment%20Formula.pdf).
4. Welte A, McWalter TA, Barnighausen T: **A Simplified Formula for Inferring HIV Incidence from Cross-Sectional Surveys Using a Test for Recent Infection**. *AIDS Res Hum Retroviruses* 2009, **25**(1):125-126.
5. Auvert B, Lissouba P, Cutler E, Zarca K, Puren A, Taljaard D: **Association of oncogenic and nononcogenic human papillomavirus with HIV incidence**. *J Acquir Immune Defic Syndr* 2010, **53**(1):111-116.
